# The endocannabinoid ARA-S facilitates the activation of cardiac Kv7.1/KCNE1 channels from different species

**DOI:** 10.1080/19336950.2024.2420651

**Published:** 2024-10-27

**Authors:** Irene Hiniesto-Iñigo, Veronika A. Linhart, Ali S. Kusay, Sara I. Liin

**Affiliations:** Department of Biomedical and Clinical Sciences, Linköping University, Linköping, Sweden

**Keywords:** Electrophysiology, IKs, KCNQ1, lipid

## Abstract

The endogenous endocannabinoid-like compound N-arachidonoyl-L-serine (ARA-S) facilitates activation of the human Kv7.1/KCNE1 channel and shortens a prolonged action potential duration and QT interval in guinea pig hearts. Hence, ARA-S is interesting to study further in cardiac models to explore the functional impact of such Kv7.1/KCNE1-mediated effects. To guide which animal models would be suitable for assessing ARA-S effects, and to aid interpretation of findings in different experimental models, it is useful to know whether Kv7.1/KCNE1 channels from relevant species respond similarly to ARA-S. To this end, we used the two-electrode voltage clamp technique to compare the effects of ARA-S on Kv7.1/KCNE1 channels from guinea pig, rabbit, and human Kv7.1/KCNE1, when expressed in *Xenopus laevis* oocytes. We found that the activation of Kv7.1/KCNE1 channels from all tested species was facilitated by ARA-S, seen as a concentration-dependent shift in the voltage-dependence of channel opening and increase in current amplitude and conductance over a broad voltage range. The rabbit channel displayed quantitatively similar effects as the human channel, whereas the guinea pig channel responded with more prominent increase in current amplitude and maximal conductance. This study suggests that rabbit and guinea pig models are both suitable for studying ARA-S effects mediated via Kv7.1/KCNE1.

## Introduction

Voltage-gated ion channels are critical for cardiac electrophysiology and function [1]. Hence, understanding how these proteins are modulated by endogenous or exogenous compounds is of importance for cardiac physiology, pathology and pharmacology. The modulatory effects of compounds are commonly assessed in a broad set of experimental models ranging from heterologous cell systems to cardiomyocytes or cardiac tissue from animals, or *in vivo* animal settings. Such different models provide mechanistic and functional insights and inform about the translational potential of findings.

To aid in the interpretation and comparison of ion channel modulatory effects in different experimental models and animal studies, it is important to know whether the ion channels *per se* from relevant animal species show comparable pharmacological profiles. Previous studies have shown that this is not always the case. For instance, transient receptor potential (TRP) channels from different species are known to respond differently to several classes of channel modulators (summarized in [2]), whereas the cardiac Kv7.1/KCNE1 channel displays species-specific inhibition mediated by protein kinase C (PKC) [3–5].

In this study, we are interested in comparing the response of the cardiac Kv7.1/KCNE1 channel from different species to N-arachidonoyl-L-serine (ARA-S), an endogenous compound linked to the endocannabinoid system which has emerged as a putative modulator of cardiovascular function. This system includes lipid compounds like 2-arachidonoyl glycerol (2-AG) and anandamide (N-arachidonoyl ethanolamine, AEA), with physiological concentrations of 2-AG ranging from 1 to 400 nM in circulation [6]. Other compounds with an arachidonic acid tail and varying head groups, such as serine for ARA-S, have also been detected in human plasma [7]. Hence, the number of compounds belonging to the endocannabinoid system has expanded over the last years, although their tissue abundance and metabolic pathways are yet to be determined. For ARA-S, the synthetic and degradation pathways are anticipated to largely follow those of the well-characterized endocannabinoid AEA, which include the actions of acyltransferases and other enzymes to generate endocannabinoids from phospholipids and fatty acid amide hydrolase (FAAH) to degrade endocannabinoids (see ref [8] for more details).

ARA-S has been shown to cause vasodilation in the cardiovascular system and modulate neuronal excitability, likely by activating the Kv7.2/Kv7.3 channel, the BK channel and the N-type Cav channel [9–11]. Moreover, ARA-S was previously reported to facilitate activation of the human Kv7.1/KCNE1 channel heterologously expressed in *Xenopus laevis* oocytes [8]. This is of potential importance for cardiac physiology and pharmacology as the Kv7.1/KCNE1 channel generates the slow component of the delayed rectifier K^+^ current (*I*_Ks_) in cardiomyocytes, which contributes to cardiomyocyte repolarization and the termination of the cardiac action potential [12,13]. Hence, the facilitated activation induced by ARA-S may have beneficial effects in pathological conditions characterized by a prolonged action potential duration or QT interval (such as in Long QT Syndrome), and could inspire drug design of pharmacological compounds with similar effects. In line with this, we previously found that ARA-S reversed a prolonged action potential duration and QT interval in the guinea pig heart [8]. However, it remains unknown whether Kv7.1/KCNE1 channels from human and guinea pig (and other species) respond similarly to ARA-S, or if there are species-dependent effects.

To address this knowledge gap, we here used electrophysiology to compare the ARA-S response of human Kv7.1/KCNE1 channels to that of Kv7.1/KCNE1 channels from two species commonly used to study the impact of *I*_Ks_; namely guinea pig and rabbit [14]. Kv7.1/KCNE1 channels from each of these species were heterologously expressed in *Xenopus laevis* oocytes, to allow for a functional side-by-side comparison in a well-controlled experimental system. We found that Kv7.1/KCNE1 channels from guinea pig and rabbit responded similarly overall to the human Kv7.1/KCNE1 channel, with clear ARA-S induced shifts in the voltage-dependence of channel opening toward more negative voltages and increases in overall conductance and current amplitude. The magnitude and concentration-dependence of the ARA-S effects were comparable in human and rabbit channels. For the guinea pig channel, the ARA-S effect on the voltage-dependence of channel opening was comparable to that of human and rabbit channels, whereas increase in overall conductance and current amplitude was more pronounced. Altogether, our data show that the ARA-S induced facilitation of Kv7.1/KCNE1 channel activation is conserved among channels from several species suggesting that cardiac tissue from human, guinea pig and rabbit could all be suitable for assessing the impact of ARA-S effects mediated via this channel. However, the larger increase in current amplitude and maximal conductance of the guinea pig channel may contribute to an enhanced ARA-S effect in guinea pig models.

## Materials and methods

### Preparation of Xenopus laevis oocytes

Isolation of *Xenopus laevis* oocytes was approved by the Linköping Animal Care and Use Committee (Permits #1941 and #14515) and conforms to national and international guidelines. All procedures conform to the guidelines from Directive 2010/63/EU of the European Parliament on the protection of animals used for scientific purposes. A total of 30 female adult (minimum 1 year of age) *Xenopus laevis* animals (from Nasco, WI, USA) underwent surgery to isolate oocytes. The frogs were anaesthetized for 15 minutes in a bath of 1.4 g/L MS-222 Sandoz (Ethyl 3-aminobenzoate methanesulfonate, Sigma Aldrich, Stockholm). The depth of anesthesia was monitored by checking for reflexes when the paws were pinched. A small incision was made on the abdomen to expose lobes of oocytes, which were collected using a surgical scissor. The small incision was closed with suture and local anesthesia (Marcain 5 mg/ml and Xylocain 2%, Distansapoteket, Stockholm) topically applied to relieve pain subsequent to surgery. At the experimental endpoint (in association with the 6th surgery), animals were sacrificed by decapitation under deep anesthesia (which was induced by the bath of 1.4 g/L MS-222 Sandoz). ARRIVE guidelines were followed. Note that experiments were performed on isolated oocytes only (no *in* *vivo* experiments were performed). Oocytes were isolated and maintained as previously described [9]. The constructs used had the following accession numbers: human Kv7.1: NM_000218; human KCNE1: NM_000219; guinea pig Kv7.1: NM_001172821.1; guinea pig KCNE1: NM_001172972.1; rabbit Kv7.1: XM_008252197.2; rabbit KCNE1: NM_001109822. Oocytes were injected with RNA for human, guinea pig or rabbit Kv7.1 and KCNE1. The amounts injected were 12.5 ng of human Kv7.1 and 7.5 ng of human KCNE1, 1.25 ng of guinea pig Kv7.1 and 1.25 ng of guinea pig KCNE1, and 0.9 ng of rabbit Kv7.1 and 0.55 ng of rabbit KCNE1. Following injection, the oocytes were generally incubated at 16°C for 1 to 2 days.

### Two-electrode voltage clamp experiments on Xenopus laevis oocytes

Two-electrode voltage clamp recordings were performed at room temperature using a Dagan CA-1B amplifier (Dagan, MN, USA) or an AxoClamp 900A amplifier (Molecular Devices, CA, USA). All chemicals are from Sigma-Aldrich (Stockholm, Sweden), unless stated otherwise. N-arachidonoyl-L-serine (ARA-S) was bought from Cayman Chemicals (MI, USA) and delivered as a stock solution of 26 mM in EtOH from the supplier. The stock solution was stored at −20°C according to supplier instructions. The control solution contained (in mM): 88 NaCl, 1 KCl, 0.4 CaCl_2_, 0.8 MgCl_2_, and 15 HEPES (with pH set to 7.4 using NaOH). For ARA-S experiments, the control solution was supplemented with indicated concentrations of ARA-S. The holding voltage was set to −80 mV. Activation curves were generally generated in steps between −80 and + 60 or +80 mV in increments of 10 mV (5-s duration). The tail voltage was set to −20 or −30 mV. Control solution or ARA-S supplemented solution was continuously perfused into the oocyte chamber at a speed of 1 mL/min using a Minipuls 3 peristaltic pump (Gilson, WI, USA). ARA-S was applied until a stable effect on current amplitude was observed, achieved after approximately 10 minutes, monitored by running an application protocol stepping from a holding voltage of −80 mV to a test voltage of 0 mV every 10 seconds. In the time-matched control experiments for rabbit and guinea pig channels, experiments were performed in a similar way as with ARA-S but with EtOH only; i.e. the first control recording was performed in control solution (no vehicle) and the subsequent recordings done with vehicle only added (i.e. EtOH but no ARA-S). The tubing system and recording chamber were cleaned between cells with 70% EtOH and distilled H_2_O.

To quantify the voltage dependence of channel opening, tail currents were measured shortly after stepping to the tail voltage and plotted against the preceding activation voltage. A Boltzmann function was fitted to the data to generate the conductance versus voltage (*G*(*V*)) curve:

(1) $$G\left(V\right)=\;G_{\mathrm{MIN}}+\left(G_{\mathrm{MAX}}-G_{\mathrm{MIN}}\right)/\;\left\{1+\exp\left[\frac{V_{50}-V}{s}\right]\;\right\},$$

where *G*_MIN_ is the minimum conductance, *G*_MAX_ the maximum conductance, *V*_50_ the midpoint (i.e. the voltage at which the conductance is half the maximal conductance determined from the fit), and *s* the slope of the curve (which was shared between the control and ARA-S in each oocyte). To calculate average *G*(*V*) curves, individual *G*(*V*) curves were first normalized relative to the predicted *G*_MAX_ value from the Boltzmann fit. The difference in steady-state current amplitude induced by the compound in each oocyte (i.e. Δ*I*_amp_) was calculated at the end of the activation pulse to 0, +20 mV or +40 mV, which are physiologically relevant voltages [15] and normalized to the current amplitude in the control solution. The effect on *G*_MAX_ or *I*_amp_ are shown in percentages (0% means no change).

To plot the concentration dependence of the compound-induced effect as a function of the compound concentration, the following concentration-response curve was fitted to the data:

(2) $$\Delta \;\mathrm{Effect}=\;\Delta \mathrm{effec}t_{\mathrm{MAX}}/\left[1+{\left(\frac{EC_{50}}{C}\right)}^{H}\right],$$

where Δeffect_MAX_ is the maximal shift in *V*_50_, change in current amplitude or change in *G*_MAX_, EC_50_ is the concentration needed to cause 50% of the maximal effect, C the concentration of ARA-S, and H is the Hill coefficient (shared value within each data set). It should be noted, however, that not all curves could be fitted with high confidence, particularly in cases where the data showed high variability or did not show a clear concentration dependence. For instance, the *G*_MAX_ effect of ARA-S in the rabbit channel could not be fitted. In such case, no fit was performed, as indicated by the term “ambiguous” in the figure legend.

### Molecular docking

The human Kv7.1 structure (PDB: 6UZZ) [16], was prepared for docking using the protein preparation wizard implemented in the Maestro software (Schrödinger Release 2023–3: Maestro, Schrödinger, LLC, New York, NY, 2023). Preparation involved filling in the missing protein loop in the S3-S4 linker (residues G219-F222) and assigning tautomeric states to titratable residues at pH = 7. ARA-S was docked into two previously identified endocannabinoid interaction sites [8]. Docking was performed using the GLIDE software [17] with 100 docking poses specified for each of the two sites and otherwise default settings. The docking poses were ranked by docking score and the top scoring docking poses are presented.

### Statistical analysis

Average values are shown as mean ± sem. The sample size varied between experimental sets (refer to figure legends), but each experiment included a minimum of 5 oocytes from 2 different frogs. One-way ANOVA followed by Dunnett’s multiple comparisons test was used to compare properties or ARA-S effects on guinea pig and rabbit channels to the human channel. One-sample t test was used to compare an effect to a hypothetical value of 0. *p* < 0.05 is considered statistically significant. Statistical analysis of data was performed using GraphPad Prism 10.

## Results

### Kv7.1/KCNE1 channels from guinea pig and rabbit showed overall comparable biophysical behaviour to the human Kv7.1/KCNE1 channel

**Figure 1.**
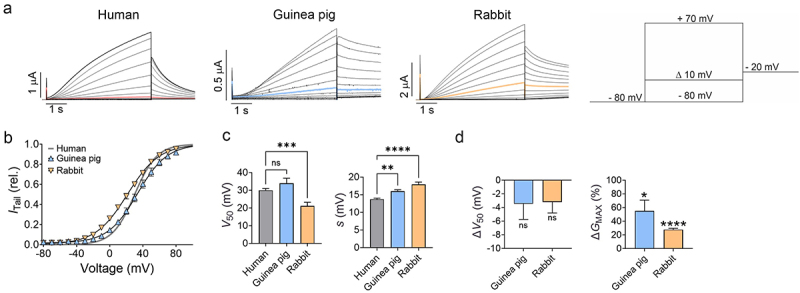
Guinea pig and rabbit Kv7.1/KCNE1 channels showed overall similar biophysical behavior to the human Kv7.1/KCNE1 channel, when expressed in *Xenopus laevis* oocytes. A) Representative currents generated by Kv7.1/KCNE1 channels from indicated species in response to the voltage protocol shown as inset. The colored traces indicate the current sweep corresponding to a 0 mV test voltage. B) average conductance *versus* voltage (*G*(*V*)) curves for indicated constructs, determined by plotting the instantaneous tail current (*I*_tail_) as a function of the preceding test voltage (see methods for details). Data shown as mean ± sem. *n* = 18 for guinea pig and 31 for rabbit. The curves represent Boltzmann fits. Best fit for guinea pig Kv7.1/KCNE1: *V*_50_ = +33.5 mV, *slope* = 17.8 mV. Best fit for rabbit Kv7.1/KCNE1: *V*_50_ = +21.4 mV, *slope* = 19.4 mV. Data for human Kv7.1/KCNE1 is included for comparison and duplicated (from [8,18,19], with *V*_50_ = +30.0 mV, *slope* = 13.4 mV. C) comparison of *V*_50_ and *slope* for indicated constructs. Data shown as mean ± sem. *n* = 152 and 30 (for *V*_50_ and *slope*, respectively) for human Kv7.1/KCNE1 (from [8,18,19] and similar to panel B for guinea pig and rabbit channels. D) time-dependent effects on *V*_50_ and *G*_MAX_ for indicated constructs (with EtOH vehicle only), during the same time-range as used to later assess ARA-S effects. Data shown as mean ± sem. *n* = 6 for guinea pig and 5 for rabbit. Statistics in panel C denote one-way ANOVA followed by Dunnett’s multiple comparisons test to compare to human. Statistics in panel D denote one-sample t test to compare to a hypothetical value of 0. * = 0.05, ** = 0.01, *** = 0.001, **** = <0.0001, ns = >0.05.

When expressed in *Xenopus laevis* oocytes, the human Kv7.1/KCNE1 channel generates slow and voltage-dependent K^+^ currents (Figure 1a), as has been shown in many previous studies [8,18,20,21]. Similarly, expression of guinea pig and rabbit Kv7.1/KCNE1 channels in *Xenopus laevis* oocytes generated slow and voltage-dependent K^+^ currents (Figure 1a). The voltage-dependence of channel opening, quantified as the half-maximal activation (*V*_50_), was similar for human and guinea pig channels, whereas the rabbit channel had a *V*_50_ shifted about −9 mV compared to the human channel (Figure 1b–c). Both the guinea pig and rabbit channels had a shallower slope of the conductance *versus* voltage relationship, compared to the human channel (Figure 1b–c). Moreover, we noted that the guinea pig channel was slower to close (Figure 1a). Altogether, guinea pig and rabbit Kv7.1/KCNE1 channels showed overall comparable, but not identical, biophysical behavior to the human channel. The behavior of the guinea pig and rabbit channels was relatively stable over time with no or minor changes to *V*_50_ in the time-range later used to assess ARA-S effects (Figure 1d), which is similar to what has been previously established for the human channel [18]. However, the guinea pig and rabbit currents gradually increased over time, reaching a + 55 ± 16% and + 28 ± 2%, respectively, increase in maximal conductance (*G*_MAX_) (Figure 1d), which needs to be taken into account when assessing pharmacological effects.

### ARA-S facilitated the activation of Kv7.1/KCNE1 from all species on the minute time scale

**Figure 2.**
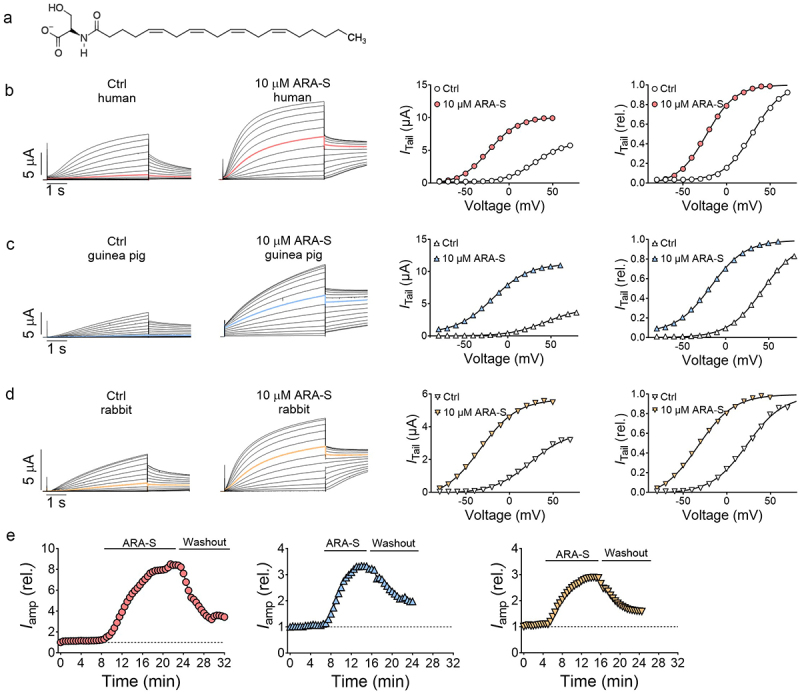
ARA-S facilitated the activation of Kv7.1/KCNE1 channels from all tested species. A) molecular structure of ARA-S. B) Representative example of traces of human Kv7.1/KCNE1 currents under control conditions and in the presence of 10 µM ARA-S and corresponding *G*(*V*) curve and normalized *I*_tail_ for better visualization of the *V*_50_ shift effect. The colored traces indicate the current sweep corresponding to a 0 mV test voltage. For this specific cell: *V*_50;ctrl_ = +30.2 mV, *I*_tailmax;ctrl_ = 6.2 µA, *V*_50;ARA-S_ = −23.0 mV, *I*_tailmax;ARA-S_ = 10.1 µA. Currents were generated in steps from − 80 to +70 mV in 10 mV steps, followed by a tail voltage of −20 mV. The holding voltage was −80 mV. C) same as in B but for guinea pig Kv7.1/KCNE1. For this specific cell: *V*_50;ctrl_ = +44.1 mV, *I*_tailmax;ctrl_ = 4.4 µA, *V*_50;ARA-S_ = −17.5 mV, *I*_tailmax;ARA-S_ = 11.2 µA. D) same as in B but for rabbit Kv7.1/KCNE1. For this specific cell: *V*_50;ctrl_ = +23.9 mV, *I*_tailmax;ctrl_ = 3.7 µA, *V*_50;ARA-S_ = −33.5 mV, *I*_tailmax;ARA-S_ = 5.7 µA. E) Representative example of the time course of the wash-in and wash-out of ARA-S effects, assessed by determining the ARA-S effect on current amplitude at 0 mV. The horizontal bar indicates when ARA-S was applied. These examples are for the application and wash-out of 10 µM ARA-S on the human (left), guinea pig (middle) and rabbit (right) Kv7.1/KCNE1 channels.

ARA-S is an endocannabinoid-like compound composed of an arachidonic tail linked to a serine head group (Figure 2a). As has been previously reported [8], extracellular application of 10 µM of ARA-S induced substantial effects on the human Kv7.1/KCNE1 channel, seen as a shifted voltage-dependence of channel opening toward more negative voltages and an increase in current amplitude and conductance over many voltages (Figure 2b). We found that 10 µM of ARA-S also induced substantial effects on guinea pig and rabbit Kv7.1/KCNE1 channels, seen as a shifted voltage-dependence of channel opening toward more negative voltages and increase in current amplitude and conductance (Figure 2c–d). The ARA-S effects occurred on the minute time scale and were partially reversible when washed out with the control solution (representative examples in Figure 2e). For the human Kv7.1/KCNE1 channel, on average 50 ± 5% of the ARA-S effect on current amplitude at 0 mV was reversed upon washout (*n* = 5, 10 µM of ARA-S).

### Kv7.1/KCNE1 from all species responded to ARA-S in a concentration-dependent manner

**Figure 3.**
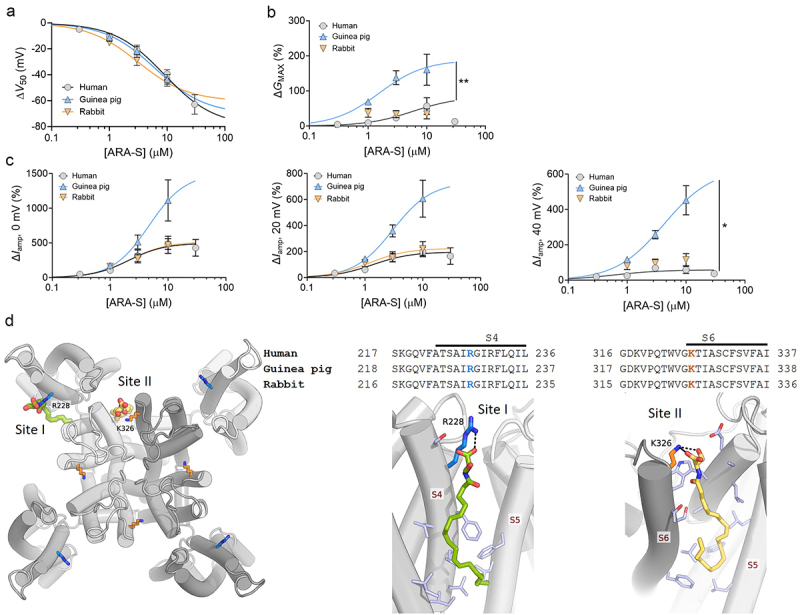
ARA-S acted on Kv7.1/KCNE1 channels from all tested species in a concentration-dependent manner. A) concentration-response relationship for the *V*_50_ effect of ARA-S on indicated constructs. Best fit for human: EC_50_ = 8.9 µM, Δ*V*_50, max_ = −79.6 mV; guinea pig: EC_50_ = 6.2 µM, Δ*V*_50, max_ = −70.4 mV; rabbit: EC_50_ = 3.1 µM, Δ*V*_50, max_ = −60.5 mV. B) same as in panel a but for the maximal conductance (*G*_MAX_). Best fit for human: EC_50_ = 5.7 µM, Δ*G*_MAX, max_ = +83%; guinea pig: EC_50_ = 1.6 µM, Δ*G*_MAX, max_ = +188%; rabbit = ambiguous. C) same as in a but for current amplitude (*I*_amp_) at indicated voltages. Best fit at 0 mV for human: EC_50_ = 2 µM, Δ*I*_amp, 0 mV, max_ = +497%; guinea pig: EC_50_ = 4.7 µM, Δ*I*_amp, 0 mV, max_ = +1499%; rabbit: EC_50_ = 2.2 µM, Δ*I*_amp, 0 mV, max_ = +515%. At 20 mV, human: EC_50_ = 1.4 µM, Δ*I*_amp, 20 mV, max_ = +197%; guinea pig: EC_50_ = 3.0 µM, Δ*I*_amp, 20 mV, max_ = +733%; rabbit: EC_50_ = 1.2 µM, Δ*I*_amp, 20 mV, max_ = +225%. At 40 mV, human: EC_50_ = 0.6 µM, Δ*I*_amp, 40 mV, max_ = +58%; guinea pig: EC_50_ = 4.2 µM, Δ*I*_amp, 40 mV, max_ = +631%; rabbit = ambiguous. Statistics in A-C denote one-way ANOVA with Dunnett´s multiple comparison test to compare the fitted curves between human and species. Only statistically significant differences are indicated, all other comparisons are non-significant (ns). * = 0.05, ** = 0.01. Data shown as mean ± sem. *n* = 5-8 for guinea pig and 6-7 for rabbit Kv7.1/KCNE1. Data for human Kv7.1/KCNE1 is included for comparison and duplicated (from [8], *n* = 8-12). D) molecular docking of ARA-S into two sites (left) in Kv7.1 (PDB: 6UZZ) [16] that have been previously determined to underlie endocannabinoid effects [8]. A predicted amino acid sequence comparison of human, guinea pig and rabbit Kv7.1 is provided for these sites. The S4 site (referred to as site I) is related to the *V*_50_ effect of ARA-S, with the key arginine R228 in human Kv7.1 marked in blue. The S6 site (referred to as site II) is related to the *G*_MAX_ effect of ARA-S, with the key lysine K326 in human Kv7.1 marked in orange. A close up of the two sites is provided with protein residues colored in light blue, except for R228 and K326. H-bond and salt bridge interactions to these residues are depicted as dashed black lines.

Kv7.1/KCNE1 from all three species responded to ARA-S with a concentration-dependent shift in *V*_50_ toward more negative voltages, which also overlapped in magnitude between the different channels (Figure 3a). About 3–9 µM of ARA-S was predicted to be required to reach 50% of the maximal effect on *V*_50_, with an anticipated maximal effect of −60 to −80 mV (see figure legend for details). As has been previously reported for the human Kv7.1/KCNE1 channel [8], the *G*_MAX_ effect of ARA-S was generally less robust than the *V*_50_ effect (Figure 3b). The rabbit Kv7.1/KCNE1 channel appeared to respond to ARA-S with an overall comparable *G*_MAX_ increase as the human channel, whereas the response of the guinea pig channel was more prominent (Figure 3b). It should be noted, though, that the guinea pig channel had a time-dependent *G*_MAX_ run-up reaching about + 55% in the time range used to assess the effect of 10 µM ARA-S (see Figure 1d), likely contributing in part to the more prominent *G*_MAX_ effect of the guinea pig channel. Together, the shifted *V*_50_ and increased *G*_MAX_ contributed to an ARA-S induced increase in current amplitude (*I*_amp_) at voltages relevant to the plateau phase [15] of the cardiac action potential (Figure 3c). For *I*_amp_ at 0, 20 and 40 mV, the human and rabbit channels responded comparably to ARA-S, whereas the guinea pig channel displayed a significantly enhanced response at 40 mV (Figure 3c).

Altogether, our side-by-side comparison of the ARA-S effects on Kv7.1/KCNE1 channels from human, guinea pig and rabbit showed that the human and rabbit channels responded in a comparable manner to ARA-S for all studied parameters. The guinea pig channel showed a comparable pharmacological response to ARA-S for *V*_50_, whereas the increase in *G*_MAX_ and *I*_amp_ was more pronounced than for the human and rabbit channels. To allow for a sequence comparison of ARA-S binding sites, we conducted molecular docking to generate binding poses for ARA-S in the human Kv7.1 structure (PDB: 6UZZ) [16]. ARA-S was docked to the two previously identified endocannabinoid interaction sites [8] (Figure 3d, left). The top scoring docking pose for ARA-S in Site I, which is near the voltage sensor S4, shows that it forms salt bridge interactions with R228 through its carboxylate head group. In Site II, which is near the S6 helix of the pore domain, the top scoring pose shows that ARA-S forms salt bridge and H-bond interactions with K326 through its carboxylate and serine head groups, respectively. In both the S4 helix segment of Site I and S6 helix segment of Site II, the three channels show 100% predicted amino acid sequence identity (Figure 3d). Given that Site I and Site II underly the *V*_50_ and *G*_MAX_ effects, respectively [8], the predicted amino acid sequence identity in both sites is in line with a clear, and largely comparable ARA-S response in the human, guinea pig and rabbit channels (Figure 3d, right).

## Discussion

This study shows that the activation of Kv7.1/KCNE1 channels from guinea pig and rabbit is facilitated by the endocannabinoid-like compound ARA-S, seen as a shifted voltage-dependence of channel opening toward more negative voltages and an increase in current amplitude and conductance. This is similar to what has been previously found for the human Kv7.1/KCNE1 channel. The concentration dependence and magnitude of ARA-S effects are directly comparable between the human and rabbit channels, whereas the guinea pig channel responds with a more prominent increase in current amplitude for certain ARA-S concentrations and voltages, and a larger increase in the maximal conductance.

The similarity in response to ARA-S among human, guinea pig and rabbit channels is consistent with them having identical predicted amino acid sequences in the interaction sites. This is also in line with largely comparable activating effects of the free fatty acid docosahexaenoic acid (DHA) on human and rabbit Kv7.1/KCNE1 channels [22]. In contrast, the fatty acid analogue linoleoyl glycine (Lin-Gly) showed impaired effects on a guinea pig-like Kv7.1/KCNE1 channel compared to the human Kv7.1/KCNE1 channel, which in part could be explained by three unconserved amino acids in the N terminus of the KCNE1 subunit (corresponding to residues 36–38 in human KCNE1) [23]. This suggests that although free fatty acids, fatty acid analogues, and endocannabinoids utilize similar interaction sites on the Kv7.1/KCNE1 channel [8,24,25], different residues may dictate the detailed response of the channel. This may be because lipophilic compounds with different chemical properties, such as acyl tail structure or head group geometry, are able to form interactions with different amino acid residues on channel proteins [24–30]. Since chemically related compounds can have species-dependent responses, it is imperative to functionally compare their effects on channels from relevant species. Moreover, despite sequence conservation in Site II, the guinea pig channel responded to ARA-S with a more prominent *G*_MAX_ increase. In part, the continuous current run-up of the construct detected in time-matched control experiments is likely a contributing factor but cannot entirely explain the enhanced *G*_MAX_ response. Hence, factors beyond the protein coding sequence in Site II and time-dependent effects must contribute. Speculatively, this may involve deviating intrinsic properties of the guinea pig channel compared to the human and rabbit channels. For instance, previous work has shown that channels with a lower open probability, and more room for improved conductance, tend to respond to activity-enhancing modulators with a more prominent *G*_MAX_ increase [30].

Altogether, our data show that Kv7.1/KCNE1 channels from guinea pig and rabbit generate typical Kv7.1/KCNE1-like currents when expressed in *Xenopus laevis* oocytes and suggest that both guinea pig and rabbit models are suitable for studying the functional impact of ARA-S mediated via Kv7.1/KCNE1. However, while the ARA-S effects via Kv7.1/KCNE1 channels themselves are anticipated to be directly comparable in human and rabbit models, ARA-S effects mediated by guinea pig Kv7.1/KCNE1 in guinea pig tissue may tend to overestimate the functional impact of ARA-S. A limitation of this study is that recordings were done at room temperature, as *Xenopus* oocytes do not tolerate body temperature. Also, it should be noted that other factors than different pharmacology of channels *per se* may contribute to varying effects in different models [2]. For instance, *I*_Ks_ has been shown to play differently important roles in cardiomyocyte repolarization depending on species and context [31]. Additionally, while ARA-S has been shown to bind only weakly to cannabinoid receptor 1 (CB1), and has been reported to have no detectable binding to CB2 and TRPV1 channels at concentrations as high as 30 µM, ARA-S is suggested to influence the phosphorylation of Akt, MAPK, and ERK1/2 signaling pathways through G-protein coupled receptor 55 (GPR55) [32,33]. Although GPR55 expression is low in cardiac cells, its presence suggests that ARA-S may have additional roles in cardiomyocytes beyond activating the Kv7.1/KCNE1 channel [34]. Finally, the uncertainty regarding the local concentrations of ARA-S in cardiomyocytes complicates our understanding of its physiological and pathophysiological roles in the human heart (while endocannabinoid concentrations may range from 1 to 400 nM, the specific levels of ARA-S remain unknown) [35]. With this in mind, it would be interesting in future work to study, side-by-side, ARA-S effects in cardiac tissue from different species.

## Data Availability

All data underlying the conclusions are provided in figures or figure legends. For any additional requests, please contact the corresponding author via e-mail (sara.liin@liu.se).
